# Growth Factor Receptor Expression in Oropharyngeal Squamous Cell Cancer: Her1–4 and c-Met in Conjunction with the Clinical Features and Human Papillomavirus (p16) Status

**DOI:** 10.3390/cancers12113358

**Published:** 2020-11-13

**Authors:** Eric Deuss, Dorothee Gößwein, Désirée Gül, Stefanie Zimmer, Sebastian Foersch, Claudia S. Eger, Ivonne Limburg, Roland H. Stauber, Julian Künzel

**Affiliations:** 1 Department of Otorhinolaryngology Head and Neck Surgery, University Hospital, 45147 Essen, Germany; 2 Department of Otorhinolaryngology Head and Neck Surgery, Molecular and Cellular Oncology, University Medical Center, 55131 Mainz, Germany; D.goesswein@gmx.de (D.G.); Desiree.Wuensch@unimedizin-mainz.de (D.G.); eger.claudia@gmx.de (C.S.E.); ilimburg@students.uni-mainz.de (I.L.); rstauber@uni-mainz.de (R.H.S.); julian.kuenzel@ukr.de (J.K.); 3 Institute of Pathology, University Medical Center, 55131 Mainz, Germany; stefanie.zimmer2@unimedizin-mainz.de (S.Z.); sebastian.foersch@unimedizin-mainz.de (S.F.); 4 Institute for Biotechnology, Shanxi University, No. 92 Wucheng Road, Taiyuan 030006, China; 5 Ear, Nose and Throat Department, University Hospital, 93053 Regensburg, Germany

**Keywords:** growth factor receptor, EGFR, c-Met, p16, oropharyngeal squamous cell cancer OPSCC, targeted therapy, immunohistochemistry, HPV

## Abstract

**Simple Summary:**

Growth factor expression is a negative prognostic factor in head and neck squamous cell carcinoma (HNSCC). Targeted therapy has a limited effect on the treatment of advanced stages due to evolving resistance mechanisms. The aim of this study was to assess the distribution of growth factor receptors in oropharyngeal squamous cell cancer (OPSCC) and evaluate their role in the context of the human papillomavirus status, prognosis and possible relevance for targeted therapy. Tissue microarrays of 78 primary OPSCC, 35 related lymph node metastasis, 6 distant metastasis and 9 recurrent tumors were manufactured to evaluate the expression of human epidermal growth factor receptor (EGFR/erbB/Her)1–4 and c-Met by immunohistochemistry. EGFR and c-Met are relevant negative prognostic factors especially in noxae-induced OPSCC. Thus, dual targeting of EGFR and c-Met could be a promising prospective target in OPSCC treatment. Frequent coexpression of assessed receptors represents a possible intrinsic resistance mechanism in targeted therapy.

**Abstract:**

This study aimed to assess the distribution of growth factor receptors in oropharyngeal squamous cell cancer (OPSCC) and evaluate their role in the context of human papillomavirus (HPV) status, prognosis and potential relevance for targeted therapy. The protein expression of human epidermal growth factor receptor (Her)1–4 and c-Met were retrospectively assessed using semiquantitative immunohistochemistry on tissue microarrays and analyzed for correlations as well as differences in the clinicopathological criteria. Her1–4 and c-met were overexpressed compared to normal mucosa in 46%, 4%, 17%, 27% and 23%, respectively. Interestingly, most receptors were coexpressed. Her1 and c-Met were inversely correlated with p16 (*p* = 0.04; *p* = 0.02). Her2 and c-Met were associated with high tobacco consumption (*p* = 0.016; *p* = 0.04). High EGFR, Her3, Her4 and c-Met expression were associated with worse overall and disease-free survival (*p* ≤ 0.05). Furthermore, EGFR and c-Met expression showed raised hazard ratios of 2.53 (*p* = 0.02; 95% CI 1.24–5.18) and 2.45 (*p* = 0.02; 95% CI 1.13–5.35), respectively. Her4 was expressed less in distant metastases than in corresponding primary tumors and was correlated to a higher T category. EGFR and c-Met are relevant negative prognostic factors in OPSCC, independent of known clinicopathological parameters. We suggest dual targeting of EGFR and c-Met as a promising strategy for OPSCC treatment.

## 1. Introduction

Head and neck squamous cell cancer (HNSCC) is the sixth most common cancer worldwide, accounting for over 430,000 deaths each year [1] Currently, the Union for International Cancer Control (UICC) staging system mainly considers clinical factors such as tumor size, nodal status and distant metastasis for estimating survival prognosis. Only human papillomavirus (HPV) status was considered as a relevant biomarker in the new 8th edition of the UICC staging system [2]. The number of HPV-positive oropharyngeal squamous cell cancer (OPSCC) cases is rising worldwide. In particular, industrial nations exhibit prevalence rates of over 50% in Northern America and 30–40% in Germany [3,4,5]. p16 expression serves as a surrogate parameter with high sensitivity and specificity for HPV positivity [6]. If other viruses, as exemplified by the current COVID-19 pandemic, also contribute to malignancy remains to be uncovered. Several other tissue factors were found to have an unfavorable influence on prognosis, but, until now, no relevant impact was observed [7]. In part, growth factor receptors (GFR) are already known to be involved in tumorigenesis, progression and, therefore, poor prognosis. The Human epithelial growth factor receptor EGFR/ErbB1, Her2/ErbB2, Her3/ErbB3, Her4/EbrB4 and c-Met are transmembrane receptor tyrosine kinases that form homo- or heterodimers after extracellular ligand binding. Dimerization causes intracellular phosphorylation of distinct tyrosine motifs. This leads to the activation of the phosphor-inositole-3-kinase (PI3K)/protein kinase B (Akt), rat sarcoma (Ras)/rapidly accelerated fibrosarcoma (Raf)/mitogen-activated protein (MAPK), phospholipase C (PLC)/protein kinase C (PKC), mechanistic target of rapamycin kinase (mTOR), signal transducer and activator of transcription 3 (STAT3), beta-catenin and Notch pathway by several binding proteins. Activation influences various cellular reactions, including apoptosis, migration, angiogenesis, growth, adhesion, differentiation and metastasis [8,9]. These complex networks include anti-apoptotic proteins, p53 and protease pathways [10,11,12,13,14]. EGFR overexpression in HNSCC is associated with poor prognosis, radiation resistance, higher recurrence rates and higher c-Met expression [15,16,17]. Her2 is also correlated with poor prognosis but shows lower rates of overexpression (39%) compared to 80–90% in EGFR [18,19]. Her3 expression was found in 13–100% of HNSCC cases, with a portion of 9–46% of overexpression [20]. Equally, membranous expression is associated with inferior overall survival (OS) [21]. Her4 expression is similarly associated with reduced overall survival and disease-free survival (DFS) [22]. Most notably, nuclear Her4 expression was correlated with a good prognosis in laryngeal cancer [23]. Therefore, the prognostic role of Her4 remains unclear. c-Met is only activated by hepatocyte growth factor (HGF), also known as scatter factor, in contrast to ErbB1, -3 and -4, which bind several ligands. c-Met can form heterodimers with representatives of the epithelial GFR family. Moreover, 33–80% of HNSCC cases express c-Met. This expression is associated with poor prognosis [24,25]. To date, cetuximab, a chimeric IgG1 monoclonal antibody against the first domain of the extracellular part of EGFR, is the only approved targeted treatment in HNSCC. However, it has limited response rates (10–15%) and is associated with less improvement in survival than newer checkpoint inhibitors (CPI) [26,27,28,29]. Therefore, Keynote 048 and Checkmate 651 trials beaconed CPI therapy as the new first-line treatment [30,31]. However, GFR still play a role in current research due to their impact on tumorigenesis and oncogene addiction theory [32]. Combined targeted treatment against GFR is, therefore, possibly able to function as an alternative in de-escalation strategies for HPV-positive OPSCC. Besides novel treatment strategies, such as smart nanoparticles, combined targeting of GFRs may be considered as an alternative in de-escalation strategies for treating HPV-positive OPSCC [33,34]. In addition, cetuximab remains relevant in second-line treatment. Newer TPEx regimen (docetaxel (Taxotere), cisplatin (Platinol) and cetuximab (Erbitux)) vs. EXTREME regimen (cisplatin, 5-fluorouracil and cetuximab) was found to improve quality of life with the same efficiency [35]. Cetuximab, in combination with nivolumab, showed an overall response rate (ORR) of 22% in CPI naïve recurrent and/or metastatic HNSCC and a complete remission rate of 2.2% [36]. The combination of monalizumab (anti-killer cell lectin-like receptor (NKG2A)-antibody) with cetuximab showed an ORR of 27.5% after first-line treatment with cisplatin and programmed cell death ligand 1 (PD-L1) inhibition [37]. This is noteworthy because monalizumab enhances cetuximab’s antibody-dependent cellular cytotoxicity by unleashing of natural killer cells and cytotoxic T cells [38]. These, partially conflicting, data stimulated us to assess the relevance of GFR protein expression in OPSCC in combination with HPV status, prognosis and possible relevance for targeted therapy.

## 2. Results

Clinical and pathological data of the study group are shown in Table 1. The median age of the collective was 58.5 years, with a range from 42 to 87 years. Moreover, 70% (55/78) were male, 33% (26/73) p16 positive, 58% (45/78) had more than 20 pack-years, 45% (33/78) were addicted to alcohol and 12% (9/78) showed locoregional recurrence. Primary tumors were slightly more locally restricted (T1/2 57% vs. T3/4 43%). In contrast, nodal status was accompanied by advanced categories (N0/1 42% vs. N2/3 58%). Altogether, more local advanced tumor staging was represented (III–IV 78% (61/78)). Treatment of primary tumors differed from a curative primary radio-(chemo-/immune-) therapy, surgery ± adjuvant therapy and induction chemotherapy with radio-(chemo-/immune-) therapy, together with salvage surgery, in contrast to a palliative concept.

### 2.1. Expression Data

**Figure 1:**
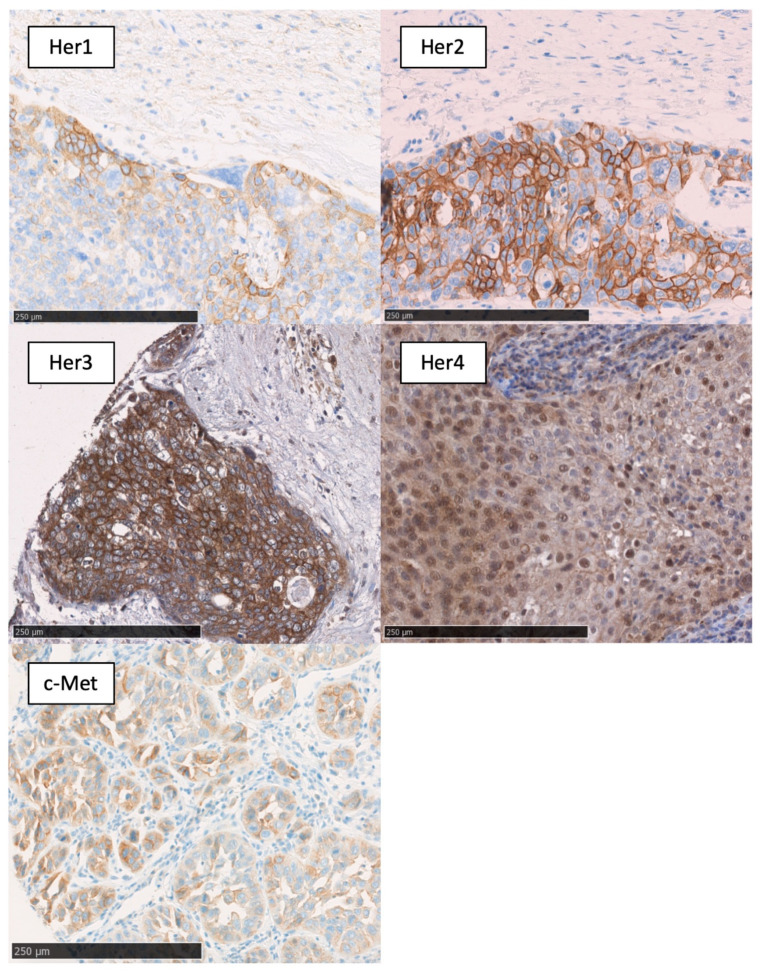
Membranous expression of EGFR; membranous expression of Her2; membranous/cytoplasmatic expression of Her3; membranous/cytoplasmatic/nuclear expression of Her4; and membranous ex-pression of c-Met. Black bar, 250 µm.

The results of immunohistochemical staining are shown in Table 2. EGFR was expressed membranous in 94% of the tumor tissue and showed overexpression compared to normal mucosa in 46% of the cases. In contrast, Her2 was expressed membranous in 15% of the cases with 4% overexpression. Her3 showed membranous and cytosolic expression patterns with the greatest difference between the amount of expression (86%) and overexpression (17%). Membranous/cytoplasmic expression of Her4 was nearly ubiquitous, with 97% and overexpression in 27% of primary tumors. On the other hand, nuclear Her4 expression was infrequent in 45% of tissue samples with 10% overexpression. c-Met was overexpressed in 23% of the cases with 46% total expression. The expression pattern is shown in Figure 1.

### 2.2. Clinicopathological Data

**Figure 2:**
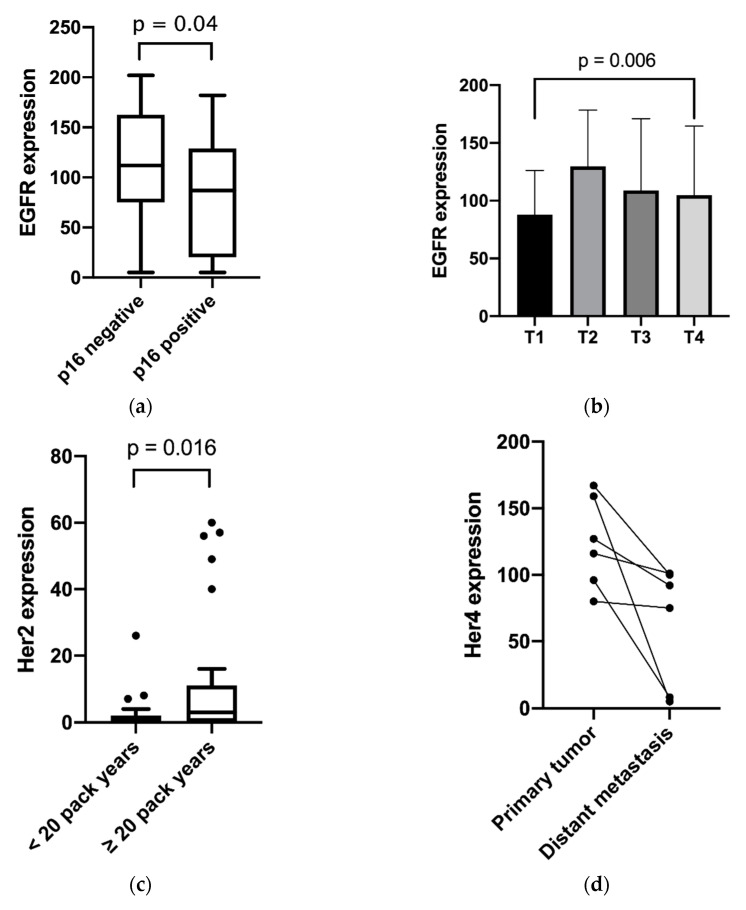

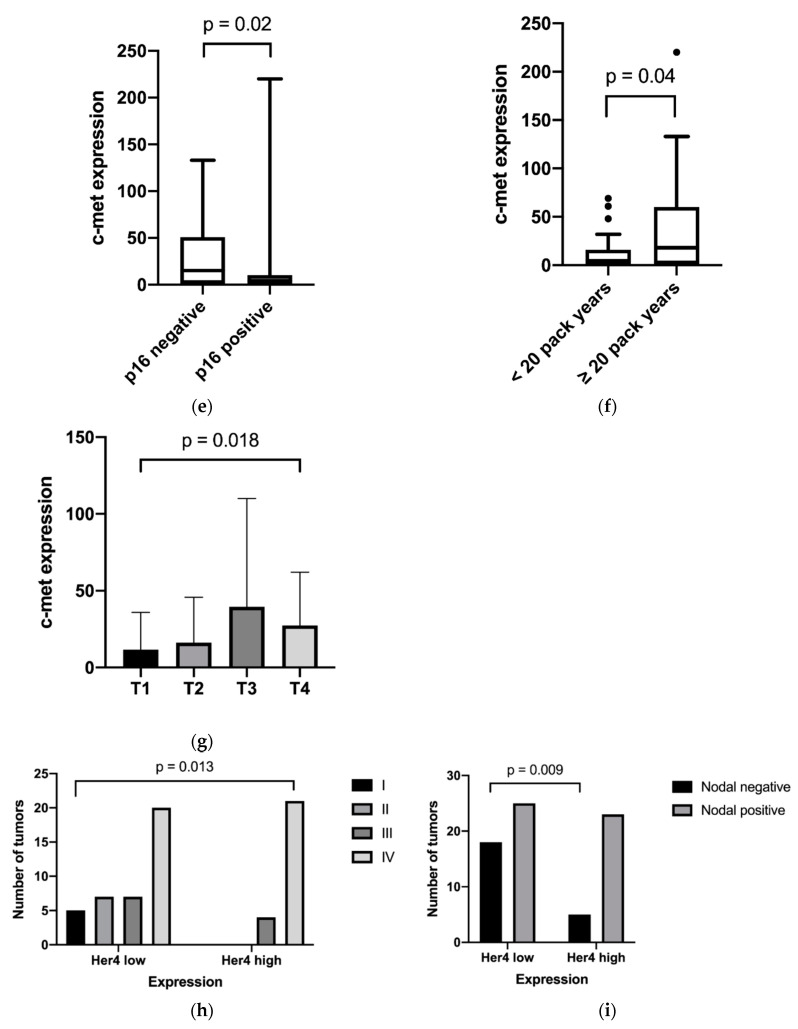
Expression distribution of growth factor receptors in relation to the clinical features: (**a**) EGFR and p16 expression; (**b**) EGFR expression and T category; (**c**) Her2 expression and pack years; (**d**) Her4 expression and tumor site; (**e**) c-Met and p16 expression; (**f**) c-Met expression and pack years; (**g**) c-Met expression and T category; (**h**) Her4 Expression and tumor stage; and (**i**) Her4 expression and nodal positivity. (**a**,**c**,**e**,**f**) Box plots with whiskers showing median, quartiles, maximum and minimum; (**b**,**g**) column bar graphs showing mean with standard deviation; (**d**) column bar graph showing before and after values; and (**h**,**i**) interleaved bars comparing absolute values.

EGFR was moderately inversely correlated with p16 (*p* = 0.04), as shown in Figure 2. In addition, EGFR was found to be associated with higher T stages (*p* < 0.01). Furthermore, the study outcome showed a higher membranous expression of Her2 in the tumor tissue of patients with more than 20 pack-years compared to nonsmokers or less than 20 pack-years (*p* < 0.01). Membranous/cytosolic expression of Her4 was associated with advanced tumor staging (*p* = 0.04) and nodal positivity (*p* < 0.01). Membranous/cytosolic Her4 expression was lower in distant metastasis than in related primary tumors (*p* = 0.04). For nuclear Her4 expression, no statistically relevant clinical association was found. High c-Met expression was correlated with several unfavorable clinical factors, such as tobacco consumption over 20 pack-years (*p* < 0.01), higher T category (*p* = 0.02), p16 negativity (*p* = 0.02) and positive distant metastasis (*p* = 0.04). For these clinical features, cytosolic/membranous Her3 expression was not found to have relevant differences. No differences were shown between receptor expression in primary tumors, and corresponding lymph node metastasis or locally recurrent tumors. The relevant results are shown in Figure 2.

**Figure 3:**
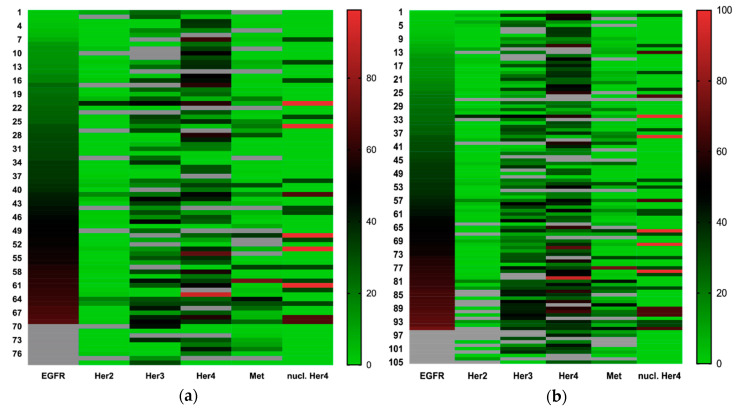
(**a**) Heatmap of growth factor receptor expression in primary tumors sorted by EGFR expression and expression values converted from a 0–300 to a 0–100 scale; and (**b**) heatmap of growth factor receptor expression in all assessed tumor tissues (primary tumor, local recurrent tumor, lymph node metastasis and distant metastasis) sorted by EGFR expression, expression values converted from a 0–300 to a 0–100 scale.

As shown in Figure 3 and Table 3, some receptors tend to be coexpressed. High Her3 and c-Met expression are associated with general high growth factor expression. Membranous/cytosolic Her4 expression showed no significant coexpression but tended to be expressed ubiquitously.

### 2.3. Survival Data

Subsequently, we examined the impact of high GFR expression on the five-year overall and disease-free survival. The univariate analysis by log-rank test showed significant differences in overall survival be-tween the high and low expression of EGFR (30% vs. 57%, *p* = 0.02), c-Met (24% vs. 62%, *p* = 0.001), nuclear Her4 (32% vs. 56%, *p* = 0.03) and membranous/cytoplasmatic Her4 (38% vs. 62%, *p* = 0.04). The statistical significance of Her2 (36% vs. 56%, *p* = 0.12) and Her3 (32% vs. 56%, *p* = 0.08) was narrowly missed. In the multivariate analysis, the influence on survival data was corrected against p16 and UICC status. Furthermore, high EGFR-(hazard ratio (HR) = 2.53, *p* = 0.01, 95% CI = 1.24–5.18) and c-Met expression (HR = 2.45, *p* = 0.02, 95% CI = 1.13–5.35) were associated with poor overall survival. Except for Her2, every receptor seemed to have a material impact on disease-free survival. Detailed data are shown in Table 4 and Table 5 as well as in Figures S1a–f and S2a–f.

## 3. Discussion

The human epithelial GFR family, including EGFR, Her2, Her3 and Her4, has been found to play an essential role in tumor growth, migration, survival, metastasis, adhesion and angiogenesis not only in HNSCC but also in glioblastoma, lung, breast, gastric and colorectal cancers [39]. Hence, various pharmaceutical treatment strategies against HNSCC have been developed. The most common strategies are tyrosine kinase inhibitors (TKIs) and monoclonal antibody treatments. Clinical trials of TKIs have failed to demonstrate significant improvement in the treatment of HNSCC. However, the use of cetuximab, a monoclonal mouse chimeric IgG1 anti-EGFR antibody, led to significant enhancement of survival and progression-free survival [40,41]. Despite the reported high EGFR expression in HNSCC, only 10–15% of tumors responded to cetuximab-containing treatment procedures. Additional treatment failure was found to occur after 2–3 months [26,27]. In particular, a better comprehension of receptor networks is needed to adapt treatment approaches regarding resistance pathways. Moreover, the number of HPV-positive OPSCC cases is rising, so its correlation to growth factor expression has to be evaluated, particularly concerning the development of de-escalation strategies in the treatment of HPV-positive OPSCC. The absence of standardized immunohistochemical staining methods and evaluation criteria leads to comparability problems. Efforts to find a consensus among critical points must be made. The methods, material and objective semiquantitative analysis criteria of this study were chosen to ensure comparability with subsequent studies.

Contrary to the reported EGFR overexpression of 80–90%, we found overexpression of 46% in primary tumors [15]. Other studies have reported even lower overexpression percentages [42]. Differences can be explained by the various staining techniques and cutoff values used. Of note, SOP for GFR analyses in HNSCCs are still missing. In addition, high ex-pression rates in 2D cell cultures were found to be not representative for 3D spherical tumor tissue [43,44]. Since high EGFR expression has not been found to influence cetuximab treatment, lower expression rates do not seem to be relevant [45,46]. However, we could prove lower EGFR expression in p16 positive OPSCC cells. High expression rates of EGFR in high tumor categories support its role in tumor progression. Lower expression of EGFR was especially found in p16 positive and small primary OPSCC. These immunohistochemical results are consistent with detected gene amplification of EGFR in HPV negative HNSCC [47]. Regarding known in vitro resistance mechanisms, we found high correlations between the coexpression of EGFR with Her3, nuclear Her4 and c-Met [48,49]. Activation of EGFR heterodimers, as well as other homodimers of epithelial GFR, can lead to resistance [50]. Furthermore, our results show a relevant negative influence of high EGFR expression on overall and disease-free survival [51,52]. Nevertheless, the findings from the sub-analysis of Bonner et al. could not be proven in the RTOG1013- and De-ESCALaTE studies [6,53]. Thus, the therapeutic relevance of cetuximab, especially in combination regimens, needs further evaluation.

Similar to EGFR, Her2 showed membranous staining only. Overexpression of Her2 is better known in other tumor types such as breast cancer, gastric cancer and salivary gland tumors [54,55]. Since the expression of Her2 in normal mucosa was nearly absent, positive tumor expression seems to be a relevant change. We found positive expression in 14% and overexpression in only 4% compared with 0–47%, as previously described [56]. Prior reports on Her2′s clinical impact are less obvious [18,57,58]. Due to different staining patterns and analysis methods, it is challenging to make comparisons. Low expression and missing benefits of TKI therapy with lapatinib support the assumption that Her2 has no important effect on prognosis. More than 20 pack-years of tobacco consumption caused higher Her2 expression in relation to clinical features compared to no tobacco consumption or less than 20 pack-years. Toxic agents seem to favor Her2 expression. The above-described correlation between p16 and Her2 expression by Pollock et al. could not be proven [56]. Likewise, a correlation between the expression of Her2 and EGFR published previously by O-Charoenrat could not be confirmed [59].

Her3 is considered to be another essential prognostic factor. Her2 and Her3 form the strongest mitogen effect by activating the PI3K/Akt pathway [60]. Nevertheless, Her3 was not found to correlate with many factors of poor prognosis in our study, in contrast to the results of Steuer et al. [61]. Only a nearly relevant negative influence on disease-free survival was found. Expression was mainly located in the cytosol and membrane, as described by Takkita et al. [21]. In particular, membranous expression rather proved to be a negative influence. The expression pattern published previously differed between cancer types and localization [61]. Our study’s expression data with 86% expression and 17% overexpression are consistent with previously described values. The large gap between expression and overexpression implies ubiquitous expression in normal tissue. Her3 showed coexpression with EGFR, Her2, c-Met and nuclear Her4. Her3′s intra-cellular domain lacks special motifs for signal activation, which depends on building heterodimers [62]. Coexpression among other receptors was shown, but it remains unclear whether heterodimerization is required.

High membranous/cytoplasmic Her4 expression was associated with higher tumor stages. Membranous/cytoplasmic Her4 expression was lower in distant metastases than in their corresponding primary tumors. Her4 showed a nearly ubiquitous expression in the cytoplasm and membrane, with a 97% ex-pression rate and 27% overexpression. Nuclear expression was rather infrequent, with 47% positive expression and 10% overexpression. This is in contrast to other studies that showed maximal expression in 55% of cases [63]. Cytoplasmic expression of Her4 in normal mucosa was only found in the basal layer expression in tumor tissue. Nuclear expression in normal mucosa was more common than in tumor tissue. There have been few studies about Her4 in HNSCC conducted, so the prognostic value is still mostly unclear [64]. Nuclear Her4 expression is considered a positive factor in cell differentiation and the promotion of apoptosis. This is mediated by the activation of Yes1 associated transcriptional regulator (YAP) and other Hippo signal pathway genes [65]. Nuclear Her4 expression is moderately correlated with the expression of all other evaluated receptors. This fact could be understood as a compensatory mechanism to tumor-promoting influences of EGFR, Her2, Her3 and c-Met. Coexpression between EGFR and nuclear Her4 suggested a negative influence on overall and disease-free survival [22]. Our membranous/cytoplasmic Her4 expression data showed no associated coexpression profile to other receptors. The coexpression of Her4 in different cell compartments demonstrated no correlation, which could be consistent with different biological functions. Likewise, findings in our OPSCC tissue samples provided no evidence that nuclear expression was a protective factor as it seems to be in laryngeal carcinoma [23]. Data from TCGA support these findings showing a loss of copy number variation (CNV) of Her4 in 39% of HNSCC tissue samples [52]. Further comparison between distinct tumor localizations is needed in order to evaluate possible differences in prognostic relevance. Nevertheless, our data demonstrate a negative association between overall and disease-free survival.

The c-Met overexpression rate of 23% is less than the previously described levels between 33% and 80% [66]. Compared to normal mucosa, tumor tissue presented stronger membranous staining than in the cytoplasm. This might highlight the relevant role of membranous expression. Clinical c-Met expression was correlated with tobacco consumption over 20 pack-years and inversely correlated with p16 expression. Inverse correlation of p16 to c-Met expression is contrary to the results of Qian et al. [67]. In non-small-cell lung carcinoma, cigarette smoking has already been found to be associated with the enhancement of oncogene addiction to c-Met [68]. High positive c-Met and p16 negative tumors are notably associated with poor prognosis [69]. This indicates a specific role for toxic induced OPSCC. Furthermore, a high c-Met expression is associated with a higher T category, confirming a poor impact on prognosis. A higher distant metastasis and lymph node metastasis rate as described by Vsiansky et al. could not be supported by our data [66]. Statistically relevant influence on poor prognosis even after adjusting for important clinical prognostic factors emphasizes its role in pathogenesis [70]. Figures S1a–f and S2a–f, which demonstrate the lowest five-year OS rate for high c-Met expression compared to other examined receptors, support this. High positive CD44, EGFR and c-Met cells have been characterized as cancer stem cells, explaining the poor prognosis of receptor coexpression in HNSCC [25,71,72]. Be-sides c-Met expression, it is possible to prevent Her2 and Her3 upregulation in HNSCC, which could explain the lower expression of Her2 and Her3 in this study [73]. c-Met seems to be a promising biomarker for prognosis in OPSCC and an attractive objective for targeted therapy. Phase 2 trial of humanized monoclonal anti c-Met antibody ficlatuzumab with or without cetuximab in cetuximab resistant recurrent or metastatic HNSCC may be able to show possible effects NCT03422536) [74]. Unfortunately, the combination of tivantinib (anti c-Met tyrosine kinase inhibitor) and cetuximab was not found to improve the response rate and survival [75]. A limitation of this study is its retrospective nature. This potentially resulted in selection bias within the study population due to differences in the treatment, tumor stage and gender. Furthermore, missing tumor tissue samples and patient files during data collection led to selection and information bias. Additionally, some missing survival data of this study population led to the loss of follow-up cases in the survival analysis. Moreover, the number of 78 patients constitutes a limitation in interpretation. These points need to be considered when discussing the results of this study.

## 4. Materials and Methods

### 4.1. Study Population

Tissue samples from 78 patients with OPSCC diagnosed between January 2010 and January 2015 in institutions of the University Medical Center of Mainz, Germany, were included in the analysis. Exclusion criteria were a history of other malignancies, as well as missing tissue and missing clinical information. Clinical and pathological data, including sex, date of birth, day of death, last contact, treatment, response evaluation criteria in solid tumors and consumption of alcohol or tobacco, were retrieved from the patients’ files. Patients were staged equally using the TNM system from the 7th Edition of UICC 2010. Furthermore, pathological data retrieved included the lymph node ratio, extranodal extension, grading, p16 expression and residual tumor status. Clinical, pathological and future immunohistochemical staining data were collected pseudonymized in a PostgreSQL database called “TuBa”. Survival data were ascertained with the aid of the Cancer Registry Rhineland Palatinate. This study was approved by the ethical review committee of the Medical Association of Rhineland Palatinate (837.485.15 (10253) 29.01.2016). All experiments were performed in accordance with relevant laws and the University Medical Center Mainz Guidelines and approved by the institutional ethics committee at the University Medical Center Mainz.

### 4.2. Tissue Microarray Construction

Tissue microarrays (TMAs) were chosen to eliminate slide to slide variation, and formalin-fixed and paraffin-embedded (FFPE) excess material was used from the tissue-biobank storage of the University Medical Center Mainz. All corresponding hematoxylin and eosin-stained slices were reviewed to ascertain the correct diagnosis and adequate tissue condition. Tumor areas were marked on slices, and 1.6-mm cores were taken from the donor paraffin block to the recipient block using a manual tissue arrayer (TMArrayer Pathology Devices, San Diego, CA, USA). Tissue cylinders were aligned and marked in a chart for identification.

### 4.3. Immunohistochemical Staining

To evaluate the expression pattern of EGFR, Her2, Her3, Her4 and c-Met in FFPE, OPSCC samples were immunohistochemically stained, as basically described previously [76,77,78,79]. Staining was performed only with antibodies that were recommended for FFPE immunohistochemistry either by the manufacturer, recently cited in publications, or routinely used clinically before. Antibody dilution was established in normal tissue, with known negative and positive expression serving as a control. Additional positive tissue samples with different expressions were used for the titration of staining intensity. Normal tissue of the oropharyngeal mucosa served as a comparison to tumor tissue expression. TMAs included muscle tissue as a negative control. TMA sections (4-µm thickness) were cut with a microtome and fixed on adhesive slides (Superfrost Plus, ThermoScientific Waltham, MA, USA) at 60 °C for 1 h. Deparaffination and rehydration were performed twice in xylol for 10 min, followed by 100%, 100%, 90%, 80% and 70% solutions of ethanol and twice distilled water each for 3 min each. Endogene peroxidase was blocked for 5 min using DAKO REAL peroxidase blocking solution (Agilent Technologies, Santa Clara, CA, USA). Antigen demasking was performed in a high or low pH DAKO target retrieval solution (Agilent Technologies) by heat-induced antigen retrieval in a steamer at 95–99 °C for 35 min. Afterwards, the sections were cooled for 10 min in Dako Wash buffer (Agilent Technologies). Dako ProteinBlock (Agilent Technologies) was used for 15 min to minimize nonspecific staining. Samples were incubated with anti-EGFR antibodies (low pH target retrieval solution (TRS); Clone E30; mouse monoclonal IgG1 Kappa; Agilent Technologies; dilution 1:50; 30 min; room temperature (RT)), anti-Her2 antibodies (low pH TRS; Hercept Test; rabbit polyclonal; Agilent Technologies; ready to use (RTU); 30 min; RT), anti-Her3 antibodies (high pH TRS; DAK-H3_IC; mouse monoclonal IgG2a Kappa; Agilent Technologies; dilution 1:50; 4 °C overnight), anti-Her4 antibodies (low pH TRS; E-AB 10231; rabbit polyclonal; Elabscience, Housten, TX, USA; dilution 1:100; 4 °C overnight). Staining was automatically performed using the Dako Autostainer Plus Slide Stainer (Agilent Technologies). Primary antibody binding was detected by incubation with horseradish-polymer conjugated secondary antibodies (DAKO REAL Envision, Agilent Technologies) for 20 min. For visualization, Dako DAB+ (Agilent Technologies) was used for 5 min. Counterstaining was performed by incubating for 1 min with DAKO REAL Hematoxylin (Agilent Technologies) and bluing in tap water for 3 min. After each step, slides were cleaned with Dako Wash buffer (tris-buffered saline (TBS)) to elute the prior reagents. Staining of c-Met was performed with anti-Met antibodies (SP44 790-4430; rabbit monoclonal; F. Hoffmann-La Roche, Basel, Switzerland) and with the standard procedure of the Ventana BenchMark Ultra system. Afterward, dehydration was performed using distilled aqua, ascendant ethanol solutions and xylol to preserve the staining results. Subsequently, embedding was performed with Enthelan (Merck, Darmstadt, Germany) and coverslips.

### 4.4. Immunohistochemical Scoring

Membranous and cytoplasmic staining were evaluated in a semiquantitative, objective and standardized manner by using the analysis software HALO (Indica Labs, Albuquerque, NM, USA) after scanning the slides with 200× magnification (Nanozoomer HT2.0, Hamamatsu Photonics, Hamamatsu, Japan). Tissue areas were classified into background, stroma, tumor and lymphocytes. Upon cell borders, the nuclear staining intensity for cell counting as well as the antigen staining intensity was adjusted to four levels: no (0), low (1+), moderate (2+), and high (3+). The analysis was not started before manual control of the classified tissue in TMA spots had taken place. In cooperation with a skilled head and neck pathologist, all of the results were screened for correctness. To create a representative score for such heterogeneous tumors as OPSCC, an already known immunohistochemical (IHC) score from 0 to 300 was chosen [80]. This was calculated by multiplying the percentage of tumor cells with their staining intensities (0–3) and summing up the results, as follows:

$$IHC_{Score}=100\times \left(1\times \frac{N\;tumor\;cells\;1+}{N\;tumor\;cells\;total}+2\times \frac{N\;tumor\;cells\;2+}{N\;tumor\;cells\;total}+3\times \frac{N\;tumor\;cells\;3+}{N\;tumor\;cells\;total}\right).$$

Nuclear Her4 staining was analyzed manually and classified in the groups 0 to 3 described above because of the large number of positive lymphocytes, resulting in a high expression of false automatically analyzed findings. For individual analysis, the expression was classified as a high or low expression using the median as the cutoff value.

Based on the applied bioinformatic software, a number of 300 different colors could not be obtained for the heatmap. We therefore decided to divide the IHC score by 3 in order to increase the clarity of color transition in the heatmap, without losing critical information.

### 4.5. Statistical Analysis

Statistical tests, charts and images were performed using SPSS version 26 (IBM, Armonk, NY, USA), Prism version 8 (GraphPad, San Diego, CA, USA) and Excel 365 (Microsoft, Redmond, WA, USA). Pearson’s chi-square test was used to evaluate the categorical clinical-pathological and expression data. Quantitative characteristics were assessed using boxplots and histograms. In addition, differences were estimated using Mann–Whitney U, Kruskal–Wallis and Wilcoxon tests. The influence of variables on survival distribution was rated using Kaplan–Meier estimates and the log-rank test for univariate subgroup analysis. Cox regression was used to evaluate the influence of prognostic factors on survival data after adjusting for multivariate established clinicopathological factors. A *p*-value of ≤ 0.05 was considered significant. Disease-free survival was defined as the period between the date of diagnosis and the date of recurrence. The latter was chosen due to the lack of information available regarding the end of treatment date.

## 5. Conclusions

EGFR and c-Met are relevant negative prognostic factors and are independent of known clinicopathological parameters in OPSCC. EGFR and c-Met expression are inversely correlated with the surrogate parameter p16. Dual inhibition of EGFR and c-Met is a promising prospective strategy in the treatment of HNSCC. Further trials of cetuximab, in combination with CPI, will reveal their therapeutic value. A pre-therapeutic immunohistochemical analysis of the growth factor panel could potentially be used to evaluate the predictive influence of coexpression data on cetuximab response and resistance. However, previous clinical data do not prove the value of EGFR as a predictive biomarker in cetuximab therapy. Membranous/cytoplasmic and nuclear Her4 expression are consistent prognostic biomarkers in OPSCC. Nevertheless, further research on the clinicopathological relevance of Her4 is required.

## Figures and Tables

**Table 1:** Clinical and pathological features of the patients.

Variables	Number (%)
Total number of patients	78 (100)
Age	
Median	58.5
Range	43–87
Gender	
Male	55 (70)
Female	23 (30)
Tumor sites	
Oropharynx	78 (100)
p16 expression	
Positive	26 (33)
Negative	47 (60)
Grading	
II	44 (56)
III	31 (40)
Smoking	
Never smoker	10 (13)
>20 pack years	45 (58)
Alcoholics	35 (45)
Recurrent tumors	9 (12)
Primary therapy	
RT/RCT/RIT	4 (5)
IC±RT/RCT/RIT±OP	24 (31)
OP±Ø/RT/RCT	47 (60)
Palliative CTx	3 (4)
T-category	
1	21 (27)
2	23 (30)
3	13 (17)
4a	20 (26)
4b	1 (1)
N-category	
0	26 (33)
1	7 (9)
2a	4 (5)
2b	21 (27)
2c	19 (24)
3	1 (1)
M-category	
0	68 (87)
1	10 (13)
UICC stage	
I	6 (8)
II	11 (14)
III	12 (15)
IVa	37 (47)
IVb	2 (3)
IVc	10 (13)

**Table 2:** Overview of expression data of EGFR, Her3, Her3, Her4 and c-Met in oropharyngeal squamous cell carcinoma.

Receptor (*N*)	Tissue	Level	*N* (%)	Receptor (*N*)	Tissue	Level	*N* (%)
membranous EGFR (70)	normal oral tissue	Mean	112	membranous Her4 (67)	normal oral tissue	Mean	127
	tumor tissue	Median	107		tumor tissue	Median	98
		Range	5–206			Range	9–276
		Expression > 10	66 (94)			Expression > 10	65 (97)
		Overexpression	32 (46)			Overexpression	18 (27)
		Low	37 (53)			Low	39 (58)
		High	33 (47)			High	28 (42)
membranous Her2 (68)	normal oral tissue	Mean	41	nuclear Her4 (78)	normal oral tissue	Mean	weak
	tumor tissue	Median	0		tumor tissue	Median	none
		Range	0–117			Range	none-strong
		Expression > 10	10 (15)			Expression	34 (45)
		Overexpression	3 (4)			Overexpression	8 (10)
		Low	44 (65)			Low	12 (15)
		High	24 (35)			High	5 (6)
membranous Her3 (66)	normal oral tissue	Mean	114	membranous c-Met (65)	normal oral tissue	Mean	37
	tumor tissue	Median	71,5		tumor tissue	Median	7
		Range	0–152			Range	0–220
		Expression > 10	57 (86)			Expression > 10	30 (46)
		Overexpression	11 (17)			Overexpression	15 (23)
		Low	35 (53)			Low	43 (66)
		High	31 (47)			High	22 (34)

**Table 3:** Bivariate correlation analysis among the growth factor receptor expression of EGFR, Her2, Her3, Her4 and c-Met.

Correlation Coefficient	Growth Factor Receptor	Analysis Parameter	EGFR	Her2	Her3	Her4	c-Met	Nuclear Her4
Spearman’s rho	EGFR	correlation coefficient	1.000	0.108	0.310 *	0.207	0.309 *	0.260 *
		sig. (2-tailed)		0.393	0.015	0.109	0.016	0.030
		*N*	70	65	61	61	60	70
	Her2	correlation coefficient	0.108	1.000	0.359 **	0.032	0.393 **	0.248 *
		sig. (2-tailed)	0.393		0.005	0.804	0,002	0.041
		*N*	65	68	59	61	59	68
	Her3	correlation coefficient	0.310 *	0.359 **	1.000	0.073	0.342 **	0.265 *
		sig. (2-tailed)	0.015	0.005		0.587	0.010	0.032
		*N*	61	59	66	57	56	66
	Her4	correlation coefficient	0.207	0.032	0.073	1.000	0.040	0.112
		sig. (2-tailed)	0.109	0.804	0.587		0.763	0.369
		*N*	61	61	57	67	59	67
	c-Met	correlation coefficient	0.309 *	0.393 **	0.342 **	0.040	1.000	0.387 **
		sig. (2-tailed)	0.016	0.002	0.010	0.763		0.001
		*N*	60	59	56	59	65	65
	nuclear Her4	correlation coefficient	0.260 *	0.248 *	0.265 *	0.112	0.387 **	1.000
		sig. (2-tailed)	0.030	0.041	0.032	0.369	0.001	
		*N*	70	68	66	67	65	78

* Correlation is significant at the 0.05 level (2-tailed). ** Correlation is significant at the 0.01 level (2-tailed).

**Table 4:** Prognostic factors on five-year overall survival from the multivariate cox regression analysis (adjustment for p16 and UICC Stages).

Receptor	Hazard Ratio (HR)	*p*-Value	95% Confidence Interval (CI)
membranous EGFR	2.53	0.01	1.24–5.18
membranous Her2	1.56	0.24	0.87–3.96
membranous Her3	1.86	0.11	0.99–4.11
membranous Her4	1.87	0.1	0.87–3.90
nuclear Her4	2.02	0.05	0.8–3.98
membranous Met	2.45	0.02	1.13–5.35

**Table 5:** Prognostic factors on five-year disease-free survival from the multivariate cox regression analysis (adjustment for p16 and UICC Stages).

Receptor	Hazard Ratio (HR)	*p*-Value	95% Confidence Interval (CI)
membranous EGFR	2.66	0.01	1.31–5.43
membranous Her2	1.72	0.024	0.84–3.53
membranous Her3	2.08	0.11	0.96–4.45
membranous Her4	2.17	0.1	1.03–4.45
nuclear Her4	2.11	0.05	1.04–4.27
membranous Met	2.50	0.02	1.15–5.42

## Supplementary Materials

The following are available online at https://www.mdpi.com/2072-6694/12/11/3358/s1, Figure S1 (a–f): Kaplan–Meier curves for prognostic impact of EGFR-, Her2-, Her3-, membranous or nuclear Her4- and c-Met expression on five-year overall survival. Figure S2 (a–f): Kaplan–Meier curves for prognostic impact of EGFR-, Her2-, Her3-, membranous or nuclear Her4- and c-Met expression on five-year disease-free survival.
