# Supplementary material for: Growth Factor Receptor Expression in Oropharyngeal Squamous Cell Cancer: Her1–4 and c-Met in Conjunction with the Clinical Features and Human Papillomavirus (p16) Status
Source: Cancers (Basel). 2020 Nov 13;12(11):3358. doi: 10.3390/cancers12113358 (PMC7697064; doi:10.3390/cancers12113358)
Supplement: Supplementary file 1 [file cancers-12-03358-s001.pdf]

# Growth Factor Receptor Expression in Oropharyngeal Squamous Cell Cancer: Her1-4 and c-met in Conjunction with the Clinical Features and Human Papillomavirus (p16) Status

**Eric Deuss, Dorothee Gößwein, Désirée Gül, Stefanie Zimmer, Sebastian Foersch, Claudia S. Eger, Ivonne Limburg, Roland H. Stauber and Julian Künzel**

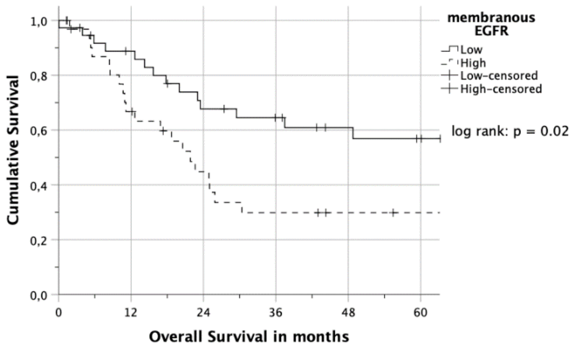


(**a**)

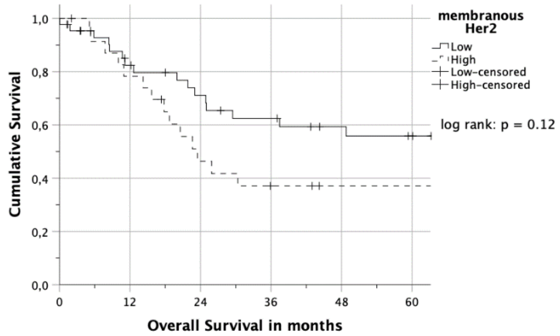


(b)

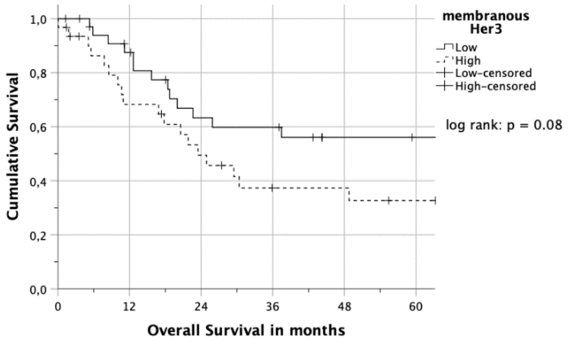


(c)

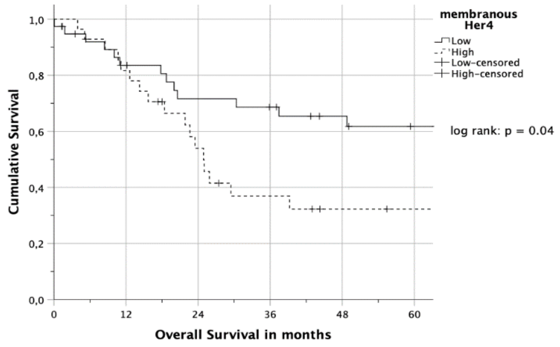


(d)

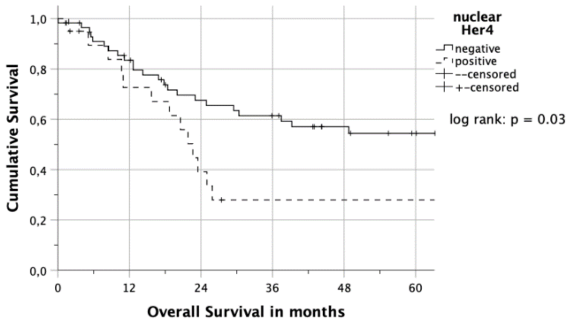


(e)

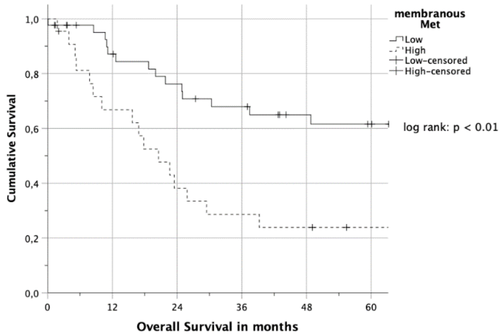


(f)

**Figure S1. (a–f):** Kaplan–Meier curves for prognostic impact of EGFR-, Her2-, Her3-, membranous or nuclear Her4- and c-Met expression on five-year overall survival.

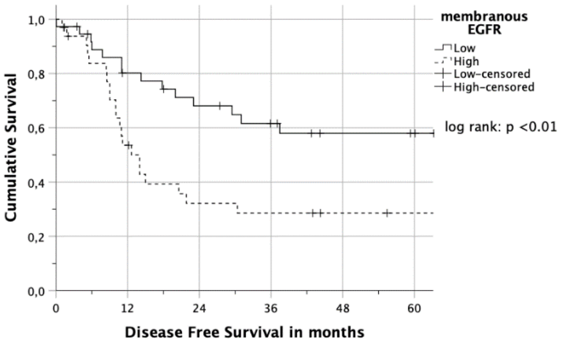


(a)

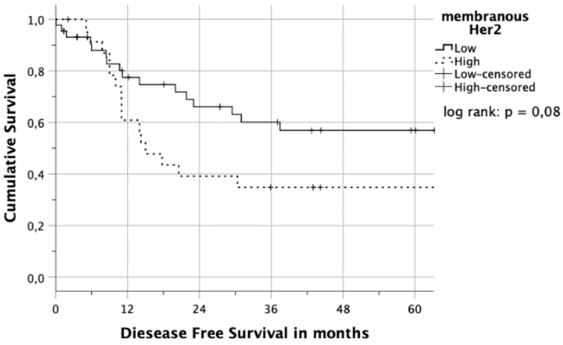


(b)

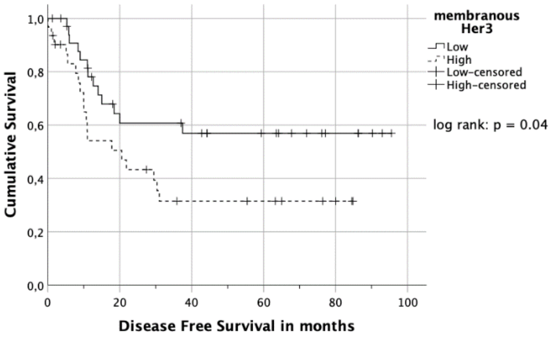


(c)

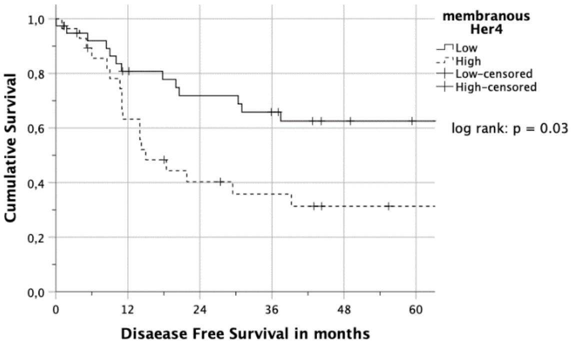


(d)

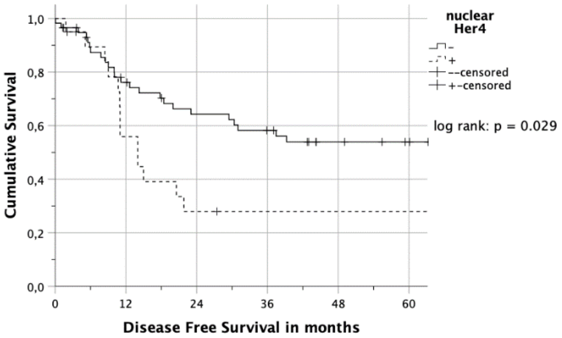


(e)

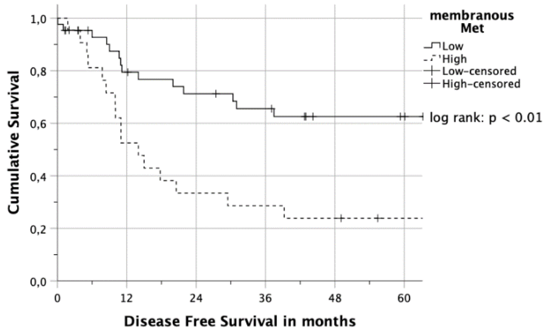


(f)

**Figure S2 (a–f):** Kaplan–Meier curves for prognostic impact of EGFR-, Her2-, Her3-, membranous or nuclear Her4- and c-Met expression on five-year disease-free survival.

© 2020 by the authors. Licensee MDPI, Basel, Switzerland. This article is an open access article distributed under the terms and conditions of the Creative Commons Attribution (CC BY) license (http://creativecommons.org/licenses/by/4.0/).
